# Respiratory Consequences of Mild-to-Moderate Obesity: Impact on Exercise Performance in Health and in Chronic Obstructive Pulmonary Disease

**DOI:** 10.1155/2012/818925

**Published:** 2012-10-14

**Authors:** Denis E. O'Donnell, Conor D. J. O'Donnell, Katherine A. Webb, Jordan A. Guenette

**Affiliations:** Respiratory Investigation Unit, Department of Medicine, Queen's University and Kingston General Hospital, 102 Stuart Street, Kingston, ON, Canada K7L 2V6

## Abstract

In many parts of the world, the prevalence of obesity is increasing at an alarming rate. The association between obesity, multiple comorbidities, and increased mortality is now firmly established in many epidemiological studies. However, the link between obesity and exercise intolerance is less well studied and is the focus of this paper. Although exercise limitation is likely to be multifactorial in obesity, it is widely believed that the respiratory mechanical constraints and the attendant dyspnea are important contributors. In this paper, we examined the evidence that critical ventilatory constraint is a proximate source of exercise limitation in individuals with mild-to-moderate obesity. We first reviewed existing information on exercise performance, including ventilatory and perceptual response patterns, in obese individuals who are otherwise healthy. We then considered the impact of obesity in patients with preexisting respiratory mechanical abnormalities due to chronic obstructive pulmonary disease (COPD), with particular reference to the effect on dyspnea and exercise performance. Our main conclusion, based on the existing and rather sparse literature on the subject, is that abnormalities of dynamic respiratory mechanics are not likely to be the dominant source of dyspnea and exercise intolerance in otherwise healthy individuals or in patients with COPD with mild-to-moderate obesity.

## 1. Introduction

The prevalence of obesity is increasing at a remarkable rate in the Western world and this has major negative health and economic ramifications [1, 2]. Obesity is clearly linked to multiple comorbidities and is an independent risk factor for reduced survival [3, 4]. Obesity is also associated with reduced activity levels [5] and this, in turn, is associated with increased risk for comorbidities which include skeletal muscle deconditioning, insulin resistance, and cardiovascular disease [6, 7]. Of interest, obese individuals who remain active appear to have lower morbidity and mortality than normal weight individuals who are sedentary [8]. A better understanding of the nature and source of exercise intolerance in obesity is required if we are to offer more effective treatment for this increasingly common health problem. The mechanisms of activity restriction in obesity are likely to be multifactorial but the role of respiratory impairment and the associated respiratory discomfort is thought to be important. This paper will focus on the respiratory factors that may influence exercise capacity in individuals with mild-to-moderate obesity based on body mass index (BMI) criteria (mild/class I (30–34.99 kg/m^2^) and moderate/class II (35–39.99 kg/m^2^)) [9, 10]. We examine how obesity affects the function of the respiratory system during the physiological stress of exercise in otherwise healthy individuals and in patients with preexisting respiratory impairment from COPD. Our paper does not include consideration of mechanisms of exercise intolerance in obese individuals who seek medical attention because of other symptoms or comorbidities, or in those with morbid obesity (class III (>40 kg/m^2^)), who may have additional abnormalities of ventilatory control.

## 2. Challenges in Obesity Research

The accurate interpretation of the existing literature on exercise physiology in obesity presents many challenges, particularly when it involves within-group comparisons of obese subjects or comparisons with age-matched normal weight individuals. While classifications of obesity by BMI are widely accepted, greater anthropometric refinement is needed to better describe obesity “phenotypes” [14]. Thus, studies that exclusively rely on BMI to define obesity can make it difficult to make definitive conclusions regarding physiological effects. Body composition (including fat free mass), fat distribution patterns, and visceral fat can be quantified by DEXA scanning [11], hydrostatic methods [15], and various radiographic imaging and bioimpedance techniques [16]. Information about fat distribution patterns (central versus peripheral) may also be inferred from calculations of waist circumference, height: weight and waist: hip ratios, and among other methods [16, 17]. 

It has been suggested that the effect of obesity on respiratory mechanics may depend to some extent on adipose tissue distribution patterns, which can vary greatly among individuals with the same BMI [18–21]. However, a recent study by Babb et al. [17] showed that the differences in respiratory mechanics (i.e., reduction in end-expiratory lung volume (EELV)) correlated as strongly with the increase in BMI as with cumulative chest wall fat or regional chest wall fat distribution patterns. 

Any study on the effect of obesity on exercise performance should also consider important confounders such as habitual activity levels (which influence fitness levels) and the possible presence of medical comorbidities (musculoskeletal, endocrine, and cardiovascular problems). A final consideration in assessing exercise performance in obesity is the exercise testing modality that is selected. The increased metabolic cost of weight-bearing exercise in obesity (e.g., walking) is amplified for a given external work rate when compared with weight-supported cycle exercise [22]. For this reason, it is possible that the obese subjects may perform better with cycle exercise compared with treadmill exercise tests, which more closely resemble daily activities. 

## 3. Cardiorespiratory Fitness in Obesity

Peak oxygen uptake (VO_2_) is widely used as a measure of aerobic capacity and cardiorespiratory fitness. Controversy still exists as to the best way to express peak VO_2_ in obesity, that is, in absolute (L/min) or relative (mL/kg/min or mL/kg fat free mass (FFM)/min) terms or as a percentage of predicted normal. Peak VO_2_ expressed in relative terms may underestimate cardiorespiratory fitness in comparison with normal weight individuals because of the higher weight denominator in obesity [23]. Lean body mass (or FFM) and skeletal muscle hypertrophy may be increased in the obese as an adaptation to the sustained mass loading effect from excessive adipose tissue [17, 24]. Lorenzo and Babb have recently suggested that peak VO_2_ should be expressed as percent predicted, rather than in absolute or relative terms, when assessing cardiorespiratory fitness in obese individuals [23]. These investigators advocated the use of the predictive VO_2_ equations of Wasserman et al. [25] for men and of Riddle et al. [26] for women. In addition to adjusting for age and height, these formulae consider ideal body weight [25, 26] and the increased metabolic cost of unloaded cycle exercise (i.e., 6 mL O_2_/min/kg of excess body weight) [25]. 

Most studies expressing peak symptom-limited VO_2_ in absolute terms or as % predicted have concluded that, contrary to expectations, cardiorespiratory fitness is generally in the normal range in individuals with mild-to-moderate obesity [11, 23, 27–29]. Peak work rate measured during incremental cycle exercise may be diminished or fall within the lower range of normal [11, 25, 30]. Other indices of cardiorespiratory fitness such as peak oxygen pulse, submaximal heart rate responses, and anaerobic/ventilatory threshold are generally within the normal range in moderate obesity [31]. The corollary is that the determinants of peak VO_2_ (i.e., cardiac output and the arteriovenous oxygen content difference) are also generally preserved in the obese. Preservation of peak symptom-limited VO_2_ also suggests that the respiratory impairment is not a proximate source of exercise limitation in otherwise healthy eucapnic obese subjects (see below). 

## 4. Respiratory Consequences of Obesity at Rest

The mass loading effects of excess adipose tissue on the chest wall and abdomen results in reduced compliance (increased stiffness) of the relaxed respiratory system [32–35]. While early physiological studies emphasized the contribution of reduced chest wall compliance [32], more recent studies in anesthetized subjects highlight the significant contribution of reduced lung compliance [35, 36]. Thus, excessive bibasal airway closure and air trapping [37], diffuse heterogeneous microatelectasis, and relatively increased intrathoracic blood volume [38] collectively increase static lung elastic recoil pressure [34]. The net effect of these obesity-related changes on lung and chest wall compliance is a resetting of the relaxation volume (functional residual capacity (FRC) or EELV) of the respiratory system to a lower volume than predicted in normal weight individuals [9, 29, 36]. Since resting EELV is lower, tidal volume becomes positioned closer to the lower nonlinear and less compliant extreme of respiratory system's sigmoid-shaped pressure-volume relation. Reduced respiratory system compliance contributes to increased work and oxygen cost of breathing in moderate obesity [39].

**Figure 1:**
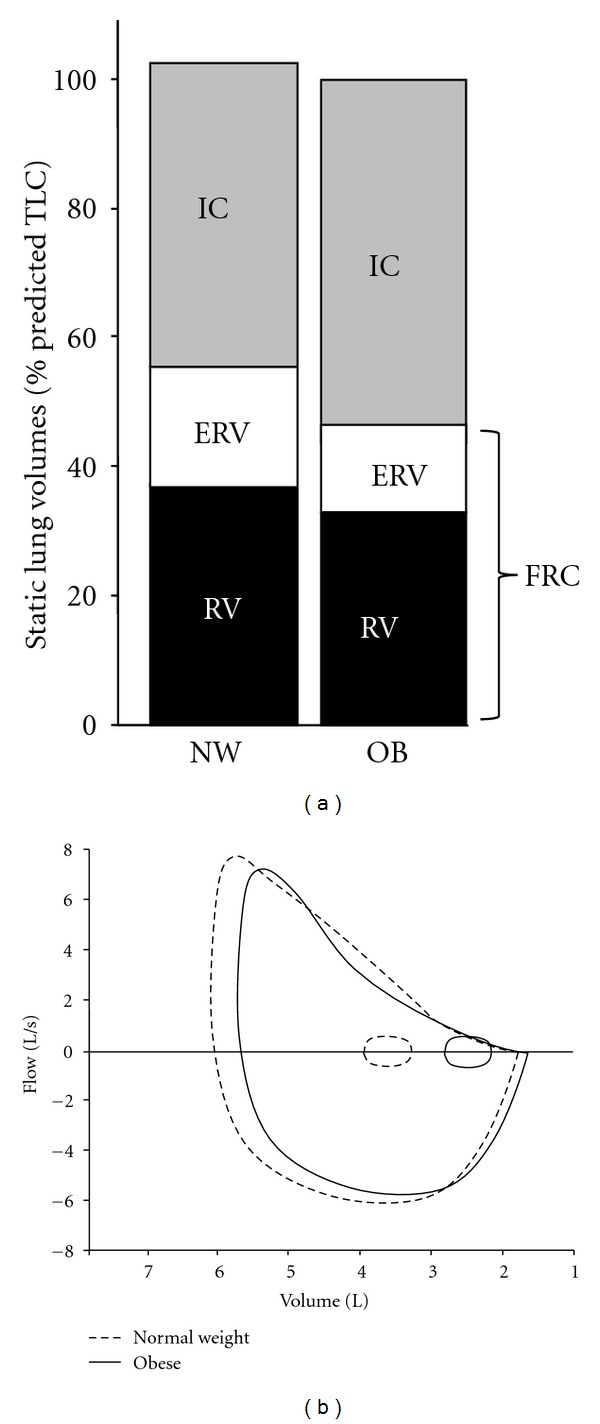
(a) Static lung volumes measured by body plethysmography are shown at rest: expiratory reserve volume (ERV) and functional residual capacity (FRC) are decreased, and inspiratory capacity (IC) is increased in the obese (OB) group compared with the normal weight (NW) group of healthy adults. (b) Maximal and tidal flow-volume loops are shown at rest in normal weight (dashed lines) and obese (solid lines) subjects. In obesity, tidal flow-volume loops are shifted rightwards and maximal midexpiratory flow rates may be reduced resulting in greater expiratory flow limitation during resting breathing. RV: residual volume.

The reduced EELV in obesity also means that the airways resistance is proportionately increased [40], in absolute terms [41], reflecting the reduced airway diameter compared with normal weight individuals. It is noteworthy that when the volume differences in health and obesity are accounted for as with measurements of specific airway resistance or specific conductance, this difference in airway resistance disappears [40, 42, 43]. In obesity, closing volume may occur at volumes above the lower EELV [37, 44–46]; thus, significant airway closure and gas trapping may occur in basal lung segments during the quiet tidal breathing cycle. The diminished expiratory reserve volume (ERV) in obesity compared with normal weight individuals means that the lung volume at the end of quiet tidal expiration (EELV) and following forced expiratory efforts (i.e., residual volume (RV)) are quite similar [9] (Figure 1).

## 5. Effect of Obesity on Pulmonary Function Measurements 

### 5.1. Lung Volumes and Spirometry

**Figure 2:**
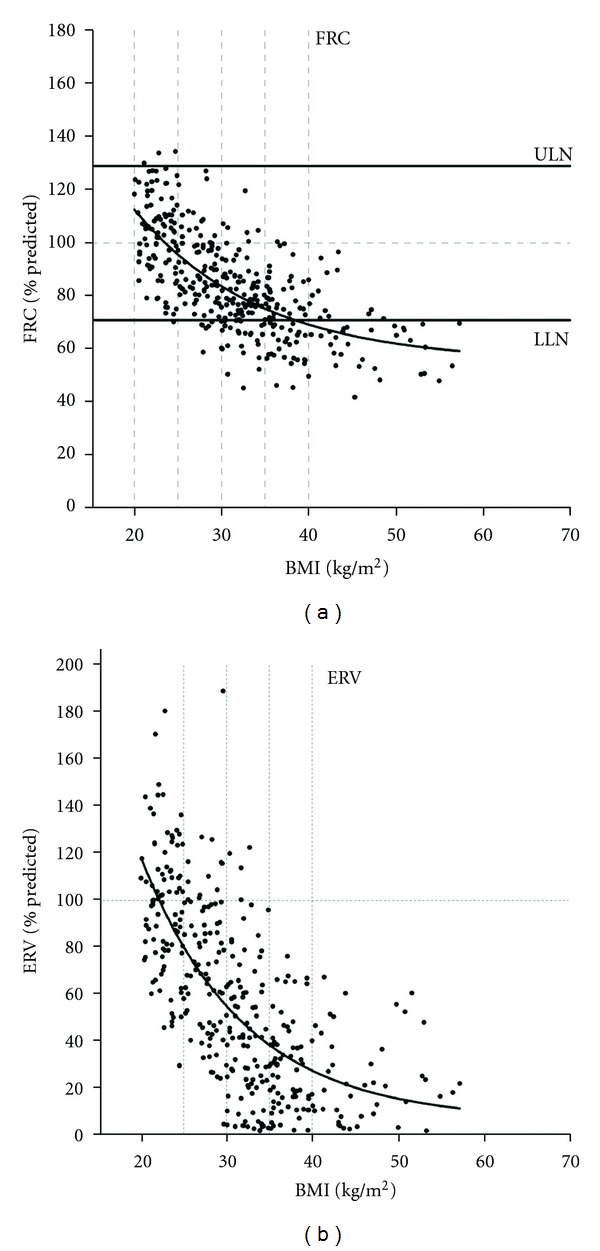
FRC and ERV decreased exponentially with increasing BMI in adult patients with normal airway function (for both regressions, *r* ^2^ = 0.49  and  *P* < 0.0001). The horizontal lines for FRC are the average upper limit of normal (ULN) and lower limit of normal (LLN) for men and women, from Jones and Nzekwu [9].

Jones and Nzekwu demonstrated an exponential relationship between increasing BMI and decreasing EELV and ERV in a healthy population [9]: these static volume components show the steepest rates of decline within the overweight and mild obesity categories (Figure 2). The decline in RV with increasing BMI is relatively less than that of EELV [9, 11] and in some studies falls within the normal range [47, 48]. Total lung capacity (TLC) may decline modestly with obesity [9, 28, 49, 50]. RV/TLC may be increased in obesity reflecting air trapping secondary to increased volume-dependent airway closure [9, 41, 51], although Jones and Nzekwu found no significant difference in this ratio between BMI groups [9]. Vital capacity (VC) may decline as BMI increases but generally into the lower normal range [9, 48, 50, 52]. However, the inspiratory capacity (IC) and the IC/TLC ratio increase with increasing the BMI reflecting the relative preservation of TLC in the presence of decreased EELV [9, 53]. 

Spirometric forced expiratory volume in 1 second (FEV_1_), which is strongly influenced by VC, is variably affected by obesity but is usually in the lower range of normal [52, 54–56]. The FEV_1_/FVC ratio is generally normal or slightly elevated [20, 40, 41, 54, 55]. Even though plethysmographically-determined airway resistance, when corrected for alveolar volume, is similar in obese and lean individuals, there is evidence of increased peripheral airway resistance in the obese. Thus, expiratory flow rates in the mid-volume VC range may be diminished in obesity reflecting volume-dependent small airway dysfunction [41, 57] (Figure 1). Expiratory flow limitation, as measured by the negative expiratory pressure technique, is present in some patients with moderate and morbid obesity during resting breathing [11, 41, 58]. Positive end-expiratory pressures have been documented in some patients with moderate obesity in the supine posture [56].

### 5.2. Pulmonary Gas Exchange

The effect of obesity on the diffusing capacity of the lung for carbon monoxide, a measure of the alveolar-capillary surface area for gas exchange, is somewhat variable but the majority of studies report normal values [33, 50, 57, 59, 60]. An increased value may reflect the increased intrathoracic blood volume in obesity [9, 50, 61, 62]. 

Pulmonary gas exchange at rest is within normal limits in most cases. Ventilation/perfusion (V/Q) inequalities may be presented (i.e., lung units with low V/Q ratios), particularly at the lung bases, and aggravated by gravity-dependent effects in the supine posture [45]. Widening of the alveolar-to-arterial O_2_ tension gradient at rest becomes clinically significant, only in those with morbid obesity [63].

### 5.3. Respiratory Muscle Function

Static strength of the inspiratory and expiratory muscles has generally been reported to be within the normal range in mild-to-moderate obesity [64, 65]. The work of breathing is increased by 3-4 fold in moderate obesity [39] and this, in turn, may serve as an intrinsic stimulus to train the respiratory muscles. Thus, static inspiratory muscle strength may be preserved or even increased, despite the restrictive mechanics of obesity. Less information is available on the mechanical efficiency and endurance of the respiratory muscles in moderate obesity. The finding of an increased O_2_ cost of breathing, relative to the mechanical work of breathing, in obesity suggests significant mechanical inefficiency as a result of excessive adipose tissue on the chest wall and abdomen [15, 66]. Respiratory muscle function may be compromised in morbid obesity and, in some studies, improves after bariatric surgery [67]. However, little is known about the effect of weight loss on the respiratory muscle function in the moderately obese. 

## 6. Ventilatory Demand and Dynamic ****Mechanical Responses during Exercise in Obesity

**Figure 3:**
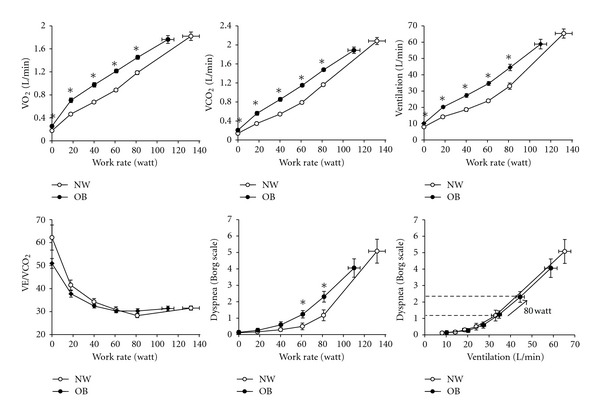
Oxygen uptake (VO_2_), carbon dioxide output (VCO_2_), minute ventilation (*V*_*E*_), the ventilatory equivalent for CO_2_ (*V*_*E*_/VCO_2_), and dyspnea intensity are shown to be relative to cycle work rate in normal weight (NW) and obese (OB) women. Relationships between dyspnea intensity and ventilation during exercise were similar in OB and NW, thus, increased dyspnea ratings at a given work rate in OB reflected the higher ventilator requirements at that work rate. Values are means ± SEM. *P < 0.05 OB versus NW at a given work rate. Data from Ofir et al. [11].

Ventilatory requirements are increased during exercise reflecting the higher metabolic cost (increased VO_2_ and VCO_2_) of external work [11, 22, 28, 68–73] (Figure 3). Despite the higher ventilatory demand, there are preliminary data to suggest that there is adequate ventilatory reserve at peak exercise in obese participants [74]. The upward parallel shift in the VO_2_/work rate slope in obesity is explained by the increased metabolic requirements of lifting heavy limbs during cycling [22, 69]. It is likely that VCO_2_ for a given power output and therefore, the ventilatory demand is higher during weight-bearing (i.e., walking) than weight-supported cycle exercise [22]. No detailed studies of pulmonary gas exchange using arterial sampling are available in individuals with mild-to-moderate obesity. Noninvasive assessments using end-tidal CO_2_ (etCO_2_) measurements in such individuals suggest that, in contrast to those with morbid obesity [75], the compensatory hyperventilation response at the end exercise is similar to that of normal weight individuals [11]. There is a little evidence to suggest that other factors known to stimulate *V*_*E*_ are more prominent in obesity compared with normal weight individuals, for example, high physiological dead space, critical arterial O_2_ desaturation, alterations in the set point for CO_2_, earlier metabolic acidosis (secondary to deconditioning), or increased metaboreceptor stimulation from the active peripheral muscles during exercise. 

**Figure 4:**
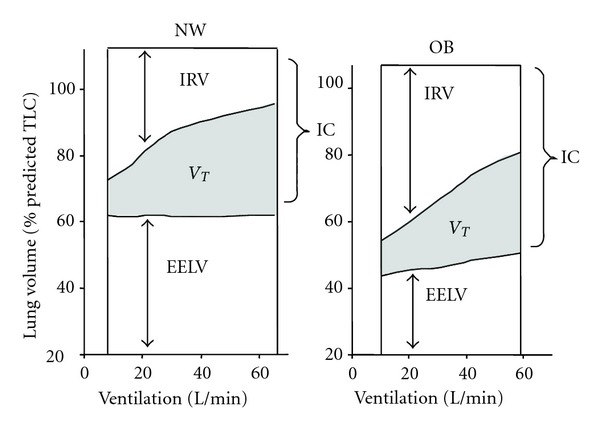
Operating lung volumes from rest-to-peak exercise are shown in normal weight (NW) and obese (OB) women. End-expiratory lung volume (EELV) increased by 0.38 L during exercise in OB but did not change in the NW subjects. Inspiratory reserve volume (IRV) was greater at rest and throughout exercise in OB women but was not statistically different at the peak of exercise. TLC: total lung capacity, IC: inspiratory capacity, *V*_*T*_: tidal volume (shaded area), from Ofir et al. [11].

Operating lung volumes and breathing pattern during cycle exercise are different in obese and normal weight individuals partly reflecting the restrictive mechanical effects of truncal and abdominal obesity [11, 28, 76]. Because EELV (and ERV) is lower at rest and throughout exercise in the obese, there is a propensity for expiratory flow limitation and increased gas trapping during the increased ventilation of exercise [11] (Figure 4). This dynamic increase in EELV may actually convey a mechanical advantage: tidal volume becomes positioned on a more compliant portion of the respiratory system's pressure-volume relation, thus avoiding the lower alinear extreme [11]. Moreover, expiratory flow limitation may be attenuated as dynamic EELV approaches the predicted relaxation volume of the respiratory system, that is, “pseudonormalization.” The dynamic increase in operating volumes, together with the naturally increased intra-abdominal pressures in obesity [47], may favorably alter the operating characteristics of the diaphragm to enhance its force-generating capacity. 

Breathing pattern responses to incremental cycle exercise are usually slightly more shallow and rapid in obese compared with normal weight individuals [11, 28, 71, 77]. The larger resting IC and inspiratory reserve volume (IRV) means that obese subjects can accommodate increases in EELV without end-inspiratory lung volume prematurely encroaching on the TLC; thus, *V*_*T*_ expansion is not more mechanically constrained during exercise compared with normal weight individuals (Figure 4). Adoption of a more rapid, shallow breathing pattern during exercise may simply be a behavioral compensatory adaptation to minimize the elastic work of breathing and attendant unpleasant respiratory sensation [78]. 

## 7. Exertional Symptoms in Obesity

Exertional symptoms may, in some cases, limit exercise performance before physiological maxima are reached and must therefore be considered in any assessment of exercise performance [31]. Intensity ratings of perceived respiratory discomfort and leg discomfort have been shown to be higher for a given external power output during cycle exercise in obese compared with normal weight subjects [11]. This suggests that mass loading of both the respiratory and peripheral skeletal muscles in the obese requires increased motor output (and contractile muscle effort) to drive these two muscle groups in tandem. The increased intensity of breathing discomfort likely reflects the increased chemostimulation and central neural respiratory drive to the respiratory muscles (and increased central corollary discharge to the somatosensory cortex) [79, 80] secondary to the relatively increased VCO_2_ for a given power output in obesity (Figure 3) [11, 81]. Babb et al. [71] have shown that increased dyspnea intensity ratings during exercise in a subgroup (37%) of women with moderate obesity was related to increased oxygen cost of breathing, measured during eucapnic voluntary hyperpnea at rest. Pulmonary function, fat distribution, peak VO_2_, and indices of respiratory mechanics, including work of breathing, were not different in the dyspneic and nondyspneic subgroups. The precise mechanistic linkage between increased dyspnea and increased O_2_ cost of breathing in this subset of obese women was not determined.

In the study of Ofir et al. [11], the dyspnea intensity/ventilation (*V*_*E*_) relation during exercise was not affected by obesity, suggesting that mechanical factors are less important in contributing to dyspnea. Thus, if increased mechanical loading of the respiratory muscles in obesity was an important contributor to dyspnea, one would anticipate that dyspnea intensity would be increased for a given *V*_*E*_ [78]. The authors have postulated that the physiological effects of obesity such as adoption of a more rapid, shallow breathing pattern (an appropriate compensation for increased elastic loading), resting IC recruitment, and “pseudonormalization” of EELVmay collectively serve to mitigate the expected rise in dyspnea intensity for a given *V*_*E*_ during exercise (Figures 3 and 4). The main conclusion of that study was that the increased dyspnea intensity for a given power output in obese individuals was primarily related to the increased ventilatory requirements and the corresponding increased central neural drive. Obesity-related abnormalities of dynamic respiratory mechanics were thought to be less important. 

## 8. Respiratory Consequences of Obesity in**** COPD

COPD, a chronic smoking-related disease of the airways, lung parenchyma, and pulmonary vasculature, is also increasing in prevalence worldwide [82]. Obesity and COPD often coexist in an increasing number of patients and this may have major implications for health care utilization [83]. Reported prevalence of obesity in COPD varies from 18% in the Netherlands [84], 25% and 27% in South America [85] and Canada [86], respectively, to as much as 54% in California [87], and may exceed obesity prevalence in the general population [84, 87, 88]. In the general population, obesity is an established risk factor for reduced life expectancy, independent of smoking status [89]. Paradoxically, epidemiological studies have shown that the patients with advanced COPD who are overweight or mildly-to-moderately obese have a survival advantage compared with underweight patients [90–92]. This “obesity paradox” has also been described in other chronic diseases (chronic heart failure, rheumatoid arthritis, and chronic renal disease) but the protective mechanisms are unknown [93]. It is noteworthy that this reduced risk of mortality was not observed in obese patients with milder COPD [91] and that subgroups of COPD patients with more severe obesity are at a greater risk of death due to respiratory failure than normal weight COPD [94]. At first glance, the imposition of the restrictive mechanical constraints of obesity on patients with preexisting expiratory flow limitation and lung hyperinflation should have detrimental effects on exercise performance, but recent studies suggest that this is not always the case (see below). 

## 9. Effects of Increasing BMI on Resting ****Pulmonary Function in COPD

**Figure 5:**
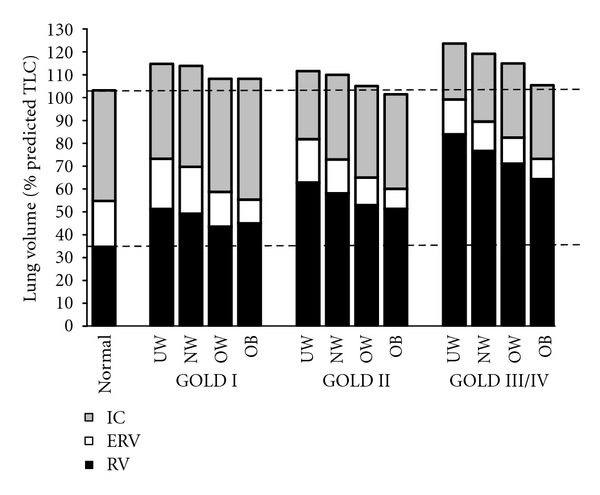
Postbronchodilator lung volume components are shown divided by global initiative for chronic obstructive lung disease (GOLD) stage and BMI. The normal column represents measurements from an age-matched healthy, nonsmoker population. UW: underweight; NW: normal weight; OW: overweight; OB: obese; IC: inspiratory capacity; ERV: expiratory reserve volume; RV: residual volume, from O'Donnell et al. [12].

In COPD, as in health, there is an exponential relation between increasing BMI and decreases in EELV and ERV [12]. This volume reduction effect occurs across all severity stages of airway obstruction and is seen even as BMI increases from normal weight to the overweight range (Figure 5). TLC and RV are relatively less affected by the increasing weight in COPD [12, 13, 95]. Importantly, as in health, the resting IC (and the IC/TLC ratio) increases in response to increasing BMI across all severity stages, reflecting the greater reduction in EELV relative to TLC. As already mentioned in relation to health [11], recruitment of IC and reduction in operating lung volumes (in absolute terms) are also potentially advantageous from a mechanical standpoint in the obese COPD patient [95]. Moreover, since a higher IC/TLC ratio (>25%) is an established favorable prognostic indicator in COPD, it is interesting to speculate that higher BMI may also be advantageous in this respect [96].

## 10. Impact of Obesity on Exercise Performance in COPD

**Figure 6:**
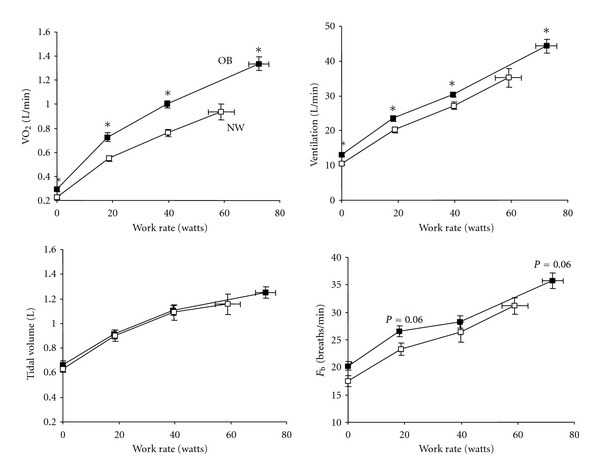
Oxygen consumption (VO_2_), ventilation, tidal volume, and breathing frequency (*F*_*b*_) are shown in response to symptom-limited cycle exercise in obese (OB) and normal weight (NW) subjects with COPD. Values are means ± SEM. *P < 0.05 OB versus NW at standardized work rates or at peak exercise, modified from Ora et al. [13].

As in health, metabolic and ventilatory requirements are elevated for a given power output during cycle exercise in obese compared with normal weight COPD patients [13, 95] (Figure 6). A recent study which compared exercise endurance time during high intensity constant work rate cycle exercise showed no differences between normal weight, overweight, and obese groups of patients with moderate-to-severe COPD [97]. In that study, patients in the overweight and obese groups had a higher peak VO_2_ in L/min than normal weight patients. Studies comparing obese with normal weight COPD groups matched for FEV_1_ found that peak VO_2_ (%predicted based on ideal body weight) during incremental cycle exercise was similar or greater in the obese [13, 95]. Additionally, there was no evidence of CO_2_ retention, based on ETCO_2_ measurements, at the sympom-limited peak of exercise. Thus, contrary to expectations, the presence of obesity could not be shown to be a disadvantage, in terms of cycle exercise capacity in COPD [13, 95].

**Figure 7:**
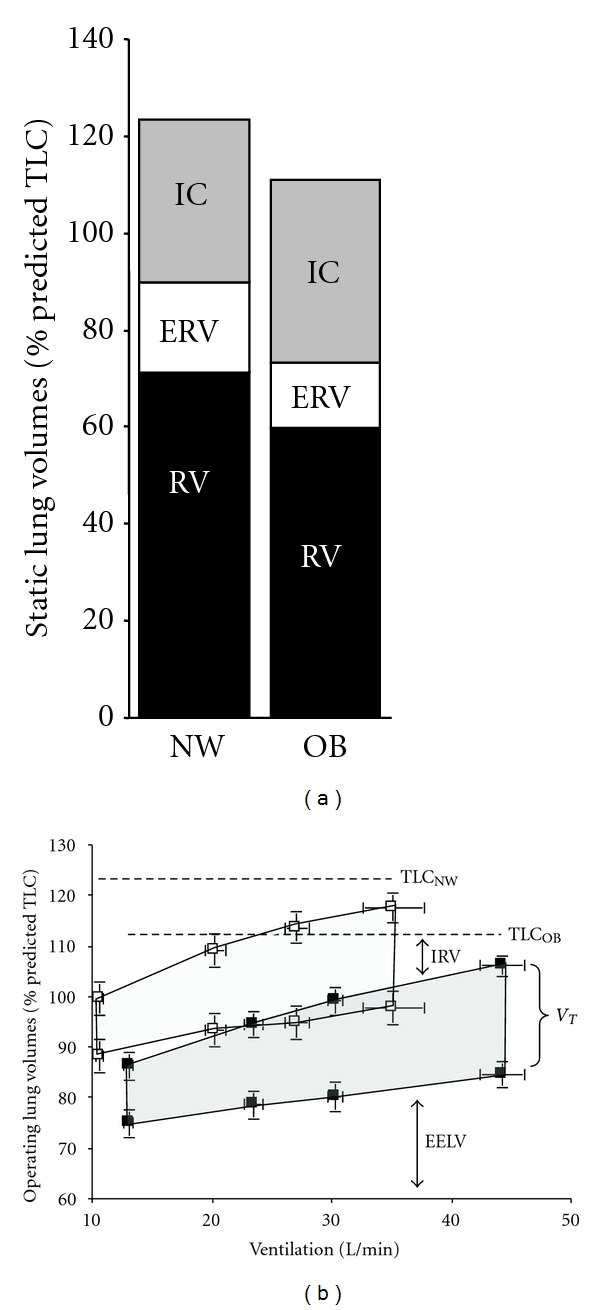
(a) Static lung volumes measured by body plethysmography are shown at rest. Expiratory reserve volume (ERV) and functional residual capacity (FRC = ERV + RV) were significantly lower in the obese (OB) group compared with the normal weight (NW) group with COPD. (b) Operating lung volumes (mean ± SEM) are shown from rest-to-peak exercise in the OB (closed symbols) and NW (open symbols) subjects: end-expiratory lung volume (EELV) was consistently lower at rest and throughout exercise in OB; the OB group reached an EELV at peak exercise that was similar to that of the NW group at the preexercise resting level. IC: inspiratory capacity; IRV: inspiratory reserve volume; *V*_*T*_: tidal volume (shaded area); RV: residual volume, from Ora et al. [13].

In COPD, the resting IC and IC/TLC ratio are important predictors of peak ventilation during symptom-limited exercise [98–100]. In patients with expiratory flow limitation, the IC represents the operating limits for *V*_*T*_ expansion during physical activity. The greater the resting lung hyperinflation, the lower the IC and, therefore, the lower the ventilation at which *V*_*T*_ reaches its plateau (or maximal value) having encroached on the minimal dynamic IRV [101]. The *V*_*T*_/*V*_*E*_ plateau, or inflection point, occurs at an IRV of 0.5–1.0 L below TLC and is an important mechanical event during exercise in COPD. This event marks the beginning of an ever widening disparity between central neural drive and the mechanical/muscular response of the respiratory system, that is, neuromechanical uncoupling [102]. At this point, dyspnea intensity escalates sharply towards intolerable levels and the distressing sensation of “unsatisfied inspiration” displaces “increased breathing effort” as the dominant qualitative descriptor [103]. The increased resting IC and IRV in obese COPD patients may mean that they can exercise to a higher *V*_*E*_ before the *V*_*T*_ inflection or plateau occurs (Figure 7) the escalation of dyspnea to intolerable levels is, therefore, delayed. 

**Figure 8:**
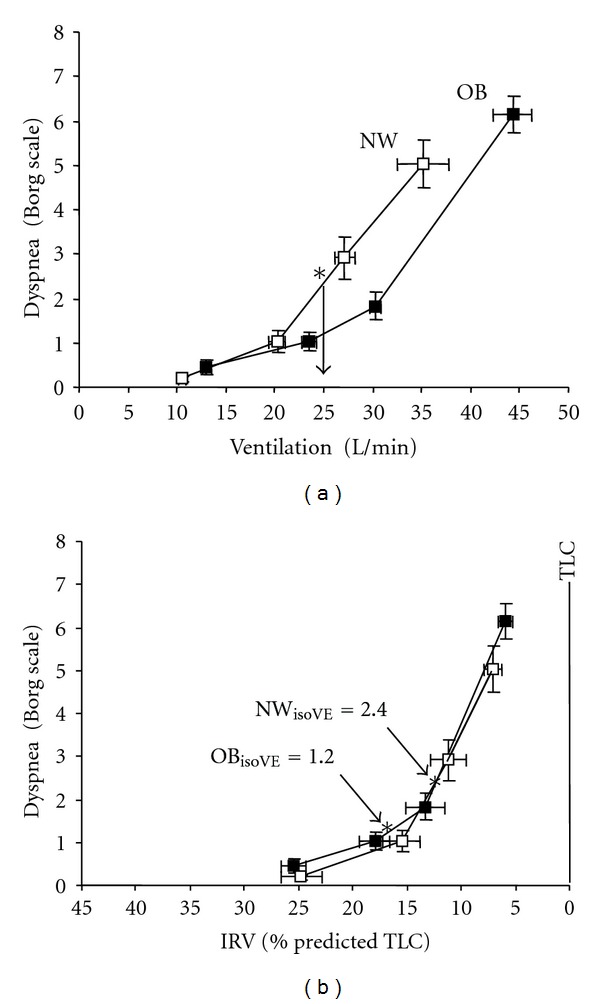
(a) Obese (OB) subjects with COPD (closed symbols) had a rightward shift in the dyspnea/ventilation relationship compared with normal weight (NW) subjects with COPD (open symbols). At an isoventilation (*V*_*E*_) of 25 L/min (vertical line with arrow), dyspnea intensity was 1.2 versus 2.4 Borg units in OB versus NW (*P < 0.01). (b) In both groups, the relationship between dyspnea intensity and inspiratory reserve volume (IRV) (standardized as a % of predicted TLC) were superimposed. At iso*V*_*E*_, OB subjects were on the flatter part of the dyspnea/IRV relation while NW subjects were on the steeper portion of the curve. Values are means ± SEM. From Ora et al. [13].

In obese COPD, dyspnea intensity ratings were not increased at any given VO_2_ or *V*_*E*_, compared with FEV_1_-matched normal weight COPD patients (Figure 8) [13, 95]. How is it possible for obese patients with COPD to accommodate the relatively higher ventilatory requirements of physical work without experiencing greater respiratory discomfort and earlier exercise limitation than normal weight COPD patients? Based on small mechanical studies, we have postulated that a number of factors may mitigate the increase in dyspnea intensity for a given *V*_*E*_ in these patients with combined restrictive-obstructive problems [13, 95]. These factors which occur in highly variable combinations include: (1) increased static elastic lung recoil pressure in obese COPD, compared with normal weight COPD, may result in larger increases in the driving pressure for tidal expiratory flows during rest and exercise; (2) increased resting IC and the lower operating lung volumes may convey mechanical advantages for the respiratory muscles, particularly the diaphragm, during exercise; (3) increased intra-abdominal pressures in obesity may also improve diaphragmatic function by forcing a more cephaloid position of this muscle at the onset of inspiration; (4) regional recruitment of lung volume (and hitherto closed airways) secondary to acute increases in EELV during exercise may attenuate the increased resistance as respired flow rates increase; (5) increased dynamic EELV may improve pulmonary gas exchange (as indicated by lower *V*_*E*_/VCO_2_ ratios) to a greater extent than in normal weight COPD patients. 

The question arises whether the presumed mechanical advantages of obesity in COPD, which preserve cycle exercise tolerance, are also applicable to weight-bearing exercise. Bautista et al. [104] showed that obese (BMI = 37 kg/m^2^) patients with COPD had reduced six minute walk distance compared with an FEV_1_-matched normal weight COPD. The mechanisms for the poorer walking performance in the obese group were not ascertained: peak VO_2_, *V*_*E*_, and cardiopulmonary responses during the tests were similar in both groups. 

Comparisons of treadmill and cycle exercise in normal weight COPD have shown greater arterial O_2_ desaturation and a higher VO_2_ for a given work rate during treadmill compared with cycle exercise [105, 106]. On the other hand, selective stress on the quadriceps muscle during cycling forces an earlier metabolic acidosis with accompanying ventilatory stimulation, which improves pulmonary gas exchange relative to treadmill exercise [105]. These differences in pulmonary gas exchange and in metabolic loading across exercise modalities may be further exaggerated in obese COPD and may influence perceptual responses during exercise, but this remains conjectural. Future treadmill-cycle comparison studies, where the increase in work rate is standardized, are needed to determine if the putative mechanical advantages of obesity in COPD during cycling are also evident during weight-bearing exercise. 

## 11. Summary

The influence of obesity on physiological and perceptual responses to exercise is an important topic, given the ever-increasing, worldwide prevalence of this condition. Contrary to expectation, there is increasing evidence that cardiorespiratory fitness, as assessed by peak symptom-limited VO_2_ (expressed as %predicted using ideal body weight), is generally preserved in otherwise healthy individuals with mild-to-moderate obesity. This preservation of exercise capacity occurs despite the presence of such obesity-related factors as mild-mechanical restriction and increased expiratory flow limitation/gas trapping, together with increased metabolic/ventilatory demands during physical exertion. We have argued that an increased IC and compensatory-breathing pattern adaptations may minimize the increased elastic work of the respiratory muscles in obesity. In turn, these factors may mitigate the expected increase in dyspnea intensity for a given ventilation during exercise in the obese. Exertional dyspnea in the obese appears to be closely related to the increased ventilatory demand and higher CO_2_ output during physical work. 

Similarly, the presence of mild-to-moderate obesity in patients with COPD appears to have little deleterious effect on peak VO_2_. Again, we have proposed that the larger IC and lower operating lung volumes throughout rest and exercise in obese COPD patients (compared with normal weight FEV_1_-matched patients) convey a mechanical advantage for the respiratory muscles. This allows obese COPD patients to accommodate the increased ventilatory requirements of a standardized physical task without experiencing greater respiratory discomfort. Collectively, these recent small physiological studies challenge the commonly held belief that critical respiratory mechanical constraints due to obesity importantly contribute to increased dyspnea and exercise intolerance in both health and disease. Future studies are needed to better elucidate the complex and multifactorial nature of daily activity restriction in obesity, particularly the interaction between pulmonary and nonpulmonary factors (e.g., metabolic and musculoskeletal abnormalities) which may be more important than previously realized.
